# Dataset of the CO_2_-rich gas emissions in the Eastern Carpathians, Romania

**DOI:** 10.1016/j.dib.2025.112376

**Published:** 2025-12-11

**Authors:** Boglárka Mercedesz Kis, Réka Szalay, Antonio Caracausi, Paolo Randazzo, Tivadar M. Tóth, László Palcsu, Judit Orsovszki, Alessandro Aiuppa, Fausto Grassa, Szabolcs Harangi

**Affiliations:** a Babeș-Bolyai University, Faculty of Biology and Geology, Department of Geology, M. Kogălniceanu str., 1, 400084 Cluj-Napoca, Romania; b Eötvös University, Institute of Geography and Earth Sciences, Department of Petrology and Geochemistry, Pázmány P. stny., 1/C, 1117 Budapest, Hungary; c Istituto Nazionale di Geofisica e Vulcanologia, Sezione di Palermo, Via Ugo La Malfa, 153, 90146 Palermo, Italy; d University of Szeged, Department of Geology, Egyetem str., 2, 6722 Szeged, Hungary; e HUN-REN Institute for Nuclear Research, Bem square, 18/C, 4026 Debrecen, Hungary; f Isotoptech Zrt., Bem square, 18/C, 4026 Debrecen, Hungary; g Università di Palermo, DiSTeM, Via Archirafi, 36, 90123 Palermo, Italy; h MTA–HUN-REN CSFK Lendület “Momentum” Pannonian Volcano Research Group, Institute for Geological and Geochemical Research, HUN-REN Research Centre for Astronomy and Earth Sciences, Budaörsi út 45, 1112 Budapest, Hungary

**Keywords:** Gas geochemistry, In-situ measurements, Multi-GAS, Gas-chromatography, Noble gases, Geogenic degassing

## Abstract

This dataset provides a comprehensive geochemical characterization of gases emitted across the Romanian segment of the Eastern Carpathians, including both volcanic and non-volcanic areas. It comprises in situ measurements of CO_2_, CH_4_, and H_2_S at 143 degassing sites, including dry vents, bubbling pools, drillings, and mineral springs, supplemented by gas-chromatographic analyses of major components (CO_2_, CH_4_, N_2_) and isotopic measurements (^3^He/^4^He, δ¹³C (CO_2_)) at 50 selected sites. The sampling strategy spans a N–S transect of the region, capturing both CO_2_- and CH_4_-rich emissions, and providing detailed coverage of dormant volcanic and non-volcanic geological settings.

Field measurements employed a portable Multi-GAS instrument with calibrated IR (for CO_2_ and CH_4_) and electrochemical (for H_2_S) sensors, ensuring high-quality in situ data. Laboratory analyses were conducted at Istituto Nazionale di Geofisica e Vulcanologia (INGV) Sezione Palermo, Italy and at HUN-REN Debrecen, Hungary, enabling robust characterization of gas compositions and isotopic ratios.

This dataset represents a high-resolution geochemical resource for the Romanian segment of the Eastern Carpathians, offering extensive information on gas compositions, noble gas signatures, and carbon isotopes. It can be reused by researchers to investigate degassing processes, gas origins, fluid migration, and tectonic controls in dormant volcanic and non-volcanic regions. Furthermore, it provides a reference for comparative studies with other global degassing systems and supports modelling of deep carbon fluxes from dormant volcanic and non-volcanic environments.

Specifications Table

**Table:** 

Subject	Earth & Environmental Sciences
Specific subject area	Geochemistry of gas emissions from dormant volcanic areas, non-volcanic areas, orogenic systems.
Type of data	Table, Raw, Processed
Data collection	A total of 158 degassing sites were surveyed in the Romanian segment of the Eastern Carpathians, between 2022 and 2023, in dry season, including dry vents, bubbling pools, drillings, and springs. In situ CO_2_, CH_4_, and H₂S were measured at 143 sites using a portable Multi-GAS instrument, equipped with IR sensors for CO_2_ (0–100 %, ±2 %) and CH_4_ (0–5 %, ±2 %), and an electrochemical H_2_S sensor (0–200 ppm, 0.25 ppm). Calibration used certified gas standards. Gas/water samples from 50 selected sites were sealed in copper tubes and underwent gas chromatographic and isotopic analyses (^3^He/^4^He, δ¹³C (CO_2_)) in laboratories from Debrecen (Hungary) and Palermo (Italy).
Data source location	Babeș-Bolyai University, Faculty of Biology and Geology, Department of Geology, Cluj-Napoca, Romania GPS coordinates for collected samples are found in Table 1. GIS-based map with the geographical locations of the sampled sites is shown in Figure 1.
Data accessibility	Specialized repository name: EarthChem Data identification number: https://doi.org/10.60520/IEDA/114173. Direct URL to data: https://ecl.earthchem.org/view.php?id=4173
Related research article	Kis, B.M., Szalay, R., Caracausi, A., Randazzo, P., Tóth, M.T., Palcsu, L., Orsovszki, J., Aiuppa, A., Grassa, F., Harangi, Sz. 2025, Geochemistry of CO2-rich gas emissions in the Carpathians: multiscale geological sources and implications for orogenic degassing, Submitted to Earth Science Reviews.

## 1 Value of the Data

The dataset provides the first comprehensive coverage of degassing sites from the Romanian segment of the Eastern Carpathians. The dataset includes 158 degassing sites, such as dry vents, bubbling pools, drillings and mineral water springs. The dataset provides major gas concentrations and isotopic ratios (^3^He/^4^He, δ¹³C (CO_2_)) following a N-S transect within the Eastern Carpathians, including both volcanic and non-volcanic regions, and CO_2_ and CH_4_-rich emissions, offering a high-resolution dataset of the Romanian segment.

Researchers can reuse this dataset to investigate degassing processes, gas origins, fluid migration and tectonic controls in dormant volcanic and non-volcanic areas. In addition, it can be used to compare Romanian sites with similar degassing systems worldwide and to support modelling of deep carbon fluxes.

## 2 Background

Recent studies aim to quantify natural carbon emissions from both volcanic and non-volcanic regions, to better understand Earth’s carbon cycle and atmospheric evolution. Studies show that diffuse CO_2_ degassing from dormant volcanic and tectonically active areas can be of similar magnitude with that from active volcanoes, linking gas fluxes to deep geological processes and regional tectonics [1,9,11,12]. Although not yet represented in global carbon budgets (e.g [12]), the Carpathians—one of Europe’s largest orogenic systems—show widespread and chemically diverse gas emissions [6,10,2]. This dataset represents the base of a study in which we investigate the geochemical composition of free gases in the Western and Eastern Carpathians to identify their sources, subsurface processes, and fluxes, emphasizing the region’s importance as a significant natural degassing area [4,5]. This data article complements the scientific article by offering details on the data acquisition process and the analytical procedures used to characterize the gas emissions of the Romanian segment of the Eastern Carpathians.

## 3 Data Description

**Fig. 1:**
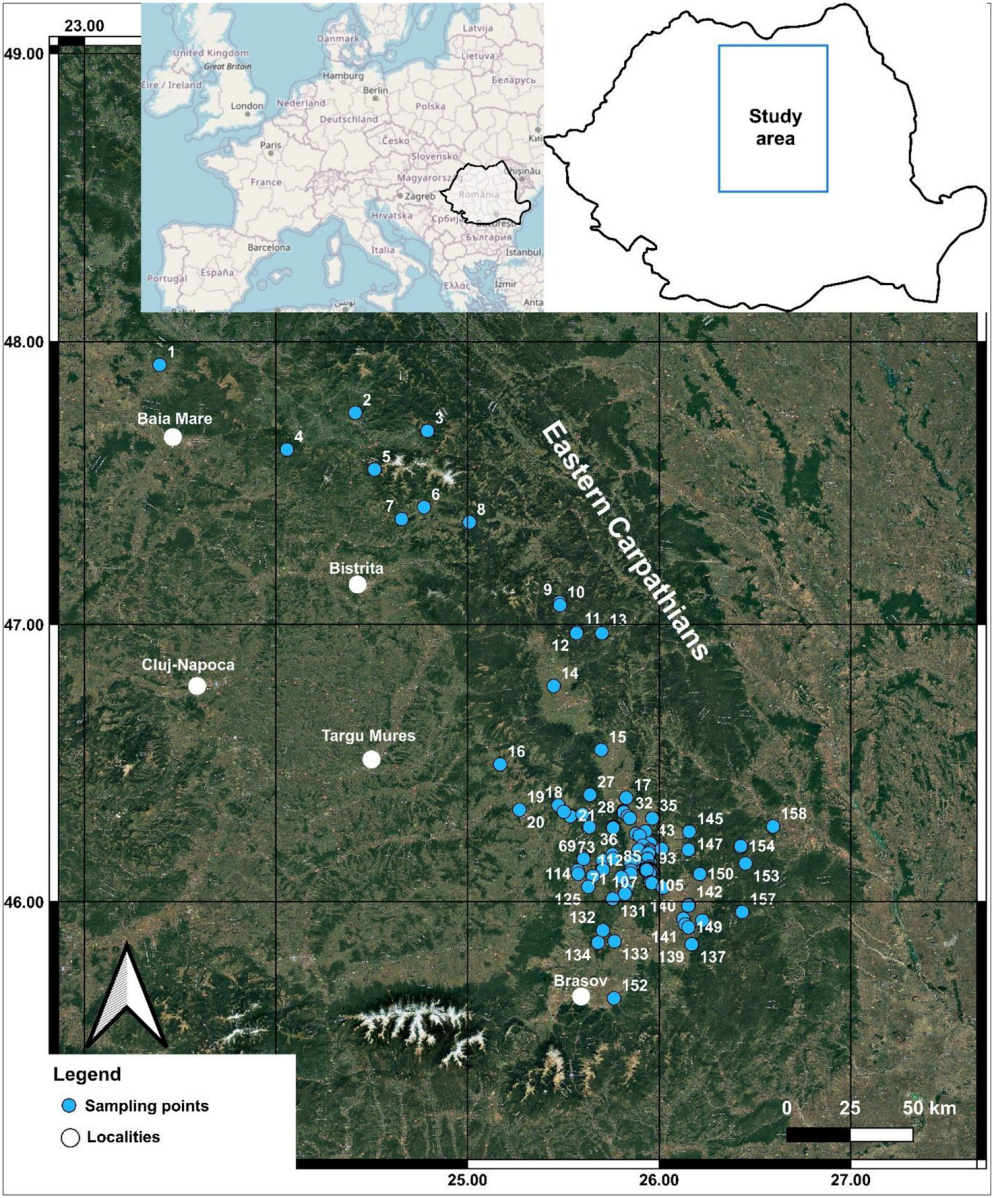
The geographical location of the sampled sites in the Romanian segment of the Eastern Carpathians on Google Earth satellite image (geographical coordinates in WGS 84).

The dataset comprises a comprehensive set of chemical and isotopic data from the Romanian segment of the Eastern Carpathians (Fig. 1). The data is accessible through the EarthChem repository with the following URL link: https://ecl.earthchem.org/view.php?id=4173.

Each sample contains an IGSN identification code that was assigned by SESAR during the uploading process of the dataset to the EarthChem repository.

The dominant gas phases of the samples are CO_2_ and CH_4_, exceeding 90 % across volcanic and non-volcanic areas and N_2_ less common.

The composition of volcanic gases reach up to 100 % CO_2_, with CH₄ from 0.01 to 7.65 %. Non-volcanic gases include pure CO_2_ and CH_4_, with CH_4_ up to 33.29 % and CO_2_ up to 42.9 %. N_2_ ranges from 0 to 96 %. Minor components include H₂S (≤560 ppm) and He (0.01–1.44 %). ^3^He/^4^He (R/R_a_) values range 0.01–4.48; δ^13^C (CO_2_) values vary between –27.2‰ and –0.05‰ (V-PDB). The dataset contains also calculated ratios of CO_2_/^3^He (Table 1).

**Table 1:** Sample names, their IGSN codes assigned by SESAR, their locations with their geographic coordinates and the build-up of the table found in the repository.

IDENTIFICATION		LOCATION		Multi-GAS data in %			GC data in %			Noble gas concentrations of free gas in %		Noble gas concentrations of dissolved gas in %			‰ (V-PDB)	
SAMPLE NAME	IGSN	LATITUDE	LONGITUDE	CO_2_	CH_4_	H_2_S	CO_2_	CH_4_	N_2_	He	Ne	He	Ne	R/R_a_	^13^C_CO2_	CO_2_/^3^He
name given by collector	unique ID assigned by SESAR	decimal degrees, negative to indicate S	decimal degrees, negative to indicate W													
EC1	10.58052/IE1180001	47.92	23.39													
EC2	10.58052/IE1180002	47.75	24.41													
EC3	10.58052/IE1180003	47.69	24.79													
EC4	10.58052/IE1180004	47.62	24.06													
EC5	10.58052/IE1180005	47.55	24.51													
EC6	10.58052/IE1180006	47.42	24.77													
EC7	10.58052/IE1180007	47.37	24.66													
EC8	10.58052/IE1180008	47.36	25.01													
EC9	10.58052/IE1180009	47.08	25.48													
EC10	10.58052/IE118000A	47.07	25.48													
EC11	10.58052/IE118000B	46.97	25.57													
EC12	10.58052/IE118000C	46.97	25.57													
EC13	10.58052/IE118000D	46.97	25.70													
EC14	10.58052/IE118000E	46.78	25.45													
EC15	10.58052/IE118000F	46.55	25.70													
EC16	10.58052/IE118000G	46.50	25.17													
EC17	10.58052/IE118000H	46.38	25.83													
EC18	10.58052/IE118000I	46.35	25.47													
EC19	10.58052/IE118000J	46.33	25.27													
EC20	10.58052/IE118000K	46.33	25.27													
EC21	10.58052/IE118000L	46.31	25.54													
EC22	10.58052/IE118000M	46.31	25.54													
EC23	10.58052/IE118000N	46.31	25.54													
EC24	10.58052/IE118000O	46.31	25.54													
EC25	10.58052/IE118000P	46.33	25.50													
EC26	10.58052/IE118000Q	46.32	25.60													
EC27	10.58052/IE118000R	46.39	25.64													
EC28	10.58052/IE118000S	46.39	25.64													
EC29	10.58052/IE118000T	46.33	25.81													
EC30	10.58052/IE118000U	46.33	25.81													
EC31	10.58052/IE118000V	46.33	25.82													
EC32	10.58052/IE118000W	46.31	25.84													
EC33	10.58052/IE118000X	46.31	25.84													
EC34	10.58052/IE118000Y	46.30	25.85													
EC35	10.58052/IE118000Z	46.30	25.96													
EC36	10.58052/IE1180010	46.27	25.76													
EC37	10.58052/IE1180011	46.27	25.76													
EC38	10.58052/IE1180012	46.27	25.76													
EC39	10.58052/IE1180013	46.27	25.76													
EC40	10.58052/IE1180014	46.26	25.93													
EC41	10.58052/IE1180015	46.25	25.88													
EC42	10.58052/IE1180016	46.24	25.90													
EC43	10.58052/IE1180017	46.21	25.95													
EC44	10.58052/IE1180018	46.20	25.92													
EC45	10.58052/IE1180019	46.19	25.95													
EC46	10.58052/IE118001A	46.19	25.89													
EC47	10.58052/IE118001B	46.18	25.95													
EC48	10.58052/IE118001C	46.18	25.95													
EC49	10.58052/IE118001D	46.18	25.95													
EC50	10.58052/IE118001E	46.18	25.95													
EC51	10.58052/IE118001F	46.18	25.95													
EC52	10.58052/IE118001G	46.18	25.95													
EC53	10.58052/IE118001H	46.18	25.95													
EC54	10.58052/IE118001I	46.18	25.95													
EC55	10.58052/IE118001J	46.18	25.95													
EC56	10.58052/IE118001K	46.18	25.95													
EC57	10.58052/IE118001L	46.18	25.95													
EC58	10.58052/IE118001M	46.14	25.95													
EC59	10.58052/IE118001N	46.14	25.95													
EC60	10.58052/IE118001O	46.14	25.95													
EC61	10.58052/IE118001P	46.16	25.94													
EC62	10.58052/IE118001Q	46.12	25.95													
EC63	10.58052/IE118001R	46.13	25.89													
EC64	10.58052/IE118001S	46.12	25.95													
EC65	10.58052/IE118001T	46.16	25.61													
EC66	10.58052/IE118001U	46.16	25.60													
EC67	10.58052/IE118001V	46.16	25.60													
EC68	10.58052/IE118001W	46.27	25.64													
EC69	10.58052/IE118001X	46.16	25.61													
EC70	10.58052/IE118001Y	46.16	25.76													
EC71	10.58052/IE118001Z	46.13	25.71													
EC72	10.58052/IE1180020	46.17	25.76													
EC73	10.58052/IE1180021	46.15	25.71													
EC74	10.58052/IE1180022	46.16	25.76													
EC75	10.58052/IE1180023	46.12	25.71													
EC76	10.58052/IE1180024	46.16	25.88													
EC77	10.58052/IE1180025	46.16	25.88													
EC78	10.58052/IE1180026	46.15	25.88													
EC79	10.58052/IE1180027	46.14	25.85													
EC80	10.58052/IE1180028	46.13	25.95													
EC81	10.58052/IE1180029	46.12	25.95													
EC82	10.58052/IE118002A	46.12	25.95													
EC83	10.58052/IE118002B	46.12	25.95													
EC84	10.58052/IE118002C	46.12	25.95													
EC85	10.58052/IE118002D	46.12	25.94													
EC86	10.58052/IE118002E	46.12	25.95													
EC87	10.58052/IE118002F	46.12	25.94													
EC88	10.58052/IE118002G	46.11	25.95													
EC89	10.58052/IE118002H	46.11	25.95													
EC90	10.58052/IE118002I	46.11	25.96													
EC91	10.58052/IE118002J	46.11	25.95													
EC92	10.58052/IE118002K	46.11	25.95													
EC93	10.58052/IE118002L	46.11	25.96													
EC94	10.58052/IE118002M	46.11	25.96													
EC95	10.58052/IE118002N	46.11	25.96													
EC96	10.58052/IE118002O	46.11	25.96													
EC97	10.58052/IE118002P	46.11	25.95													
EC98	10.58052/IE118002Q	46.11	25.95													
EC99	10.58052/IE118002R	46.11	25.95													
EC100	10.58052/IE118002S	46.11	25.95													
EC101	10.58052/IE118002T	46.11	25.95													
EC102	10.58052/IE118002U	46.11	25.95													
EC103	10.58052/IE118002V	46.11	25.95													
EC104	10.58052/IE118002W	46.11	25.95													
EC105	10.58052/IE118002X	46.11	25.95													
EC106	10.58052/IE118002Y	46.12	25.93													
EC107	10.58052/IE118002Z	46.12	25.93													
EC108	10.58052/IE1180030	46.12	25.93													
EC109	10.58052/IE1180031	46.12	25.93													
EC110	10.58052/IE1180032	46.12	25.94													
EC111	10.58052/IE1180033	46.11	25.85													
EC112	10.58052/IE1180034	46.10	25.85													
EC113	10.58052/IE1180035	46.11	25.57													
EC114	10.58052/IE1180036	46.10	25.58													
EC115	10.58052/IE1180037	46.09	25.65													
EC116	10.58052/IE1180038	46.09	25.65													
EC117	10.58052/IE1180039	46.09	25.80													
EC118	10.58052/IE118003A	46.07	25.95													
EC119	10.58052/IE118003B	46.07	25.95													
EC120	10.58052/IE118003C	46.07	25.95													
EC121	10.58052/IE118003D	46.07	25.95													
EC122	10.58052/IE118003E	46.07	25.95													
EC123	10.58052/IE118003F	46.07	25.96													
EC124	10.58052/IE118003G	46.05	26.02													
EC125	10.58052/IE118003H	46.05	25.63													
EC126	10.58052/IE118003I	46.05	25.83													
EC127	10.58052/IE118003J	46.03	25.82													
EC128	10.58052/IE118003K	46.03	25.82													
EC129	10.58052/IE118003L	46.03	25.82													
EC130	10.58052/IE118003M	46.03	25.82													
EC131	10.58052/IE118003N	46.01	25.76													
EC132	10.58052/IE118003O	45.90	25.71													
EC133	10.58052/IE118003P	45.86	25.77													
EC134	10.58052/IE118003Q	45.85	25.68													
EC135	10.58052/IE118003R	45.85	26.17													
EC136	10.58052/IE118003S	45.85	26.17													
EC137	10.58052/IE118003T	45.84	26.17													
EC138	10.58052/IE118003U	45.85	26.17													
EC139	10.58052/IE118003V	45.85	26.17													
EC140	10.58052/IE118003W	45.94	26.13													
EC141	10.58052/IE118003X	45.92	26.14													
EC142	10.58052/IE118003Y	45.99	26.15													
EC143	10.58052/IE118003Z	45.99	26.15													
EC144	10.58052/IE1180040	46.19	26.01													
EC145	10.58052/IE1180041	46.25	26.16													
EC146	10.58052/IE1180042	46.19	26.15													
EC147	10.58052/IE1180043	46.25	26.16													
EC148	10.58052/IE1180044	45.93	26.23													
EC149	10.58052/IE1180045	45.91	26.15													
EC150	10.58052/IE1180046	46.10	26.21													
EC151	10.58052/IE1180047	46.10	26.21													
EC152	10.58052/IE1180048	46.65	25.76													
EC153	10.58052/IE1180049	46.14	26.45													
EC154	10.58052/IE118004A	46.20	26.43													
EC155	10.58052/IE118004B	46.20	26.42													
EC156	10.58052/IE118004C	46.20	26.43													
EC157	10.58052/IE118004D	45.96	26.43													
EC158	10.58052/IE118004E	46.27	26.59													

## 4 Experimental Design, Materials and Methods

A total of 158 degassing sites were investigated in the Romanian segment of the Eastern Carpathians, between 2022–2023 in dry seasons, including dry gas vents, bubbling pools, drillings, and mineral springs.

### 4.1 In situ measurements

In situ measurements were conducted at 143 sites using a portable Multi-GAS instrument designed for low-temperature, CO_2_-rich emissions. The Multi-GAS system, assembled at the University of Palermo for Babeș-Bolyai University, featured two IR spectrometers (Gascard NG II, Edinburgh Sensors, UK) for CO₂ (0–100 %, ±2 %) and CH_4_ (0–5 %, ±2 %), and an electrochemical sensor (T3H CiTiCeL, City Technology Ltd., UK) for H₂S (0–200 ppm, 0.25 ppm resolution). Calibration was performed at the University of Palermo using ad-hoc gas mixtures obtained by diluting certified standards of 100 vol % CO_2_, 10 vol % CH_4_, and 136 ppmv H₂S with air. Tests made with gas standards in the laboratory allowed inferring accuracy and precision at ± 5 % for CO_2_ and CH_4_, and ± 10 % for H_2_S.

In the field, gas effluents were drawn through the Multi-GAS inlet using a Boxer S-series pump (12 V, 1.8 L min⁻¹), continuously passing through the in-series sensors. Output signals were logged in real time on a CR6 datalogger (Campbell Scientific). Two filters—an external Pall Acro 50 (1 µm) and an internal 0.45 µm filter—protected the sensors from dust and moisture; the external filter was replaced regularly, and the internal one during annual calibration. Real-time control was enabled via Wi-Fi and the Logger Link app (Campbell Scientific). Measurements at each site lasted several minutes until CO_2_, CH_4_, and H₂S concentrations stabilized; average values were computed in Excel. Both dry mofettes and bubbling pools were investigated to assess performance in contrasting environments. Tests made in the laboratory after field work found that the original (pre-field) calibration could be reproduced within ±10 %. The reproducibility of the data was also tested during seasonal measurements performed at selected sites within the study area [3].

### 4.2 Gas and water sampling

Gas and water samples were collected in sealed copper tubes with pinch-off steel clamps for laboratory analyses, following the procedures described in [7,8], in a closed system, to avoid air contamination.

Fifty sites were selected for detailed chemical and isotopic characterization.

### 4.3 Analyses of major gas components with gas chromatography

Major gas components (CO_2_, CH_4_, N_2_) were analysed at the Istituto Nazionale di Geofisica e Vulcanologia (INGV) Sezione Palermo, Palermo, Italy, using an Agilent 7890B gas chromatograph equipped with an additional micro gas chromatographic module (Inficon Fusion) assembled by SRA Instrument. Once injected in the inlet system, the sample was split into two aliquots. The first aliquot was directed to the micro gas chromatographic module, for the analysis of CH_4_ and CO_2_ (and also of C_2_H6, C_3_H_8_, H_2_S, SO_2_, if present in the sample). The micro gas chromatographic module was equipped with a Poraplot U capillary column (27.5 m x 0.53 mm x 20μm) with He (≥99.9996 %) as a carrier gas, and the oven temperature was kept constant at 80 °C. The second aliquot filled the 100μl loop and injected into the column of the Agilent 7890B GC system using a 10-way Valco pneumatic valve for the quantitative analysis of N_2_ (and also, He, H_2_, O_2_, CO). The chromatographic separation was achieved with an 80 °C isothermal column CP Molsieve 5A (25 m x 0.53 mm x 50 μm) using ultra-high purity Argon (≥99.9995 %) as the carrier gas.

N_2_ and CO_2_ were detected by using a Thermal Conductivity Detector (TCD), while a Flame Ionization Detector (FID) is used for CH_4_. Detection limits were 1μmol mol^−1^ for CH_4_ and 100μmol mol^−1^ both for N_2_ and for CO_2_.

Quantitative analyses were performed using an external calibration procedure based on a three-point calibration curve. At the beginning of each working day, three mix gas standards were analyzed. These standards contained all the species at different concentration levels, A linear regression curve for each individual gas species was established by plotting the analyte peak area of the three mixed standards against their corresponding known concentrations. The concentration of a gas species in an unknown sample was subsequently determined by measuring its peak area and interpolating it in the calibration curve equation. Analytical errors were <3 % for N_2_, CO_2_ and better than 5 % for CH_4_.

### 4.4 Noble gas and δ¹³C (CO_2_) analyses

Compositional and isotopic analyses of noble gases (^3^He/^4^He) and δ^13^C(CO_2_) were conducted at the HUN-REN Institute for Nuclear Research, Debrecen, Hungary. Measurements used Helix SFT and VG5400 noble gas mass spectrometers, and a Thermo Finnigan Delta PLUS XP isotope ratio mass spectrometer. Noble gases were separated cryogenically: major gases were adsorbed at 25 K, He and Ne at 10 K, with sequential desorption at 42 K and 90 K. The analyses were calibrated with well-known air aliquots, and normalized with tiny admission of pure helium (enriched in ^3^He) and neon, as fast calibrations [8].

To analyse δ¹³C (CO_2_), CO_2_ gas was cryogenically removed from the gas samples using liquid nitrogen and measured by a Thermo Finnigan Delta PLUS XP isotope ratio mass spectrometer. Ion current ratios were first normalized to that of a working gas, and then calibrated against international carbonate standards after phosphoric-acid digestion [7]. The isotope ratios are given as δ permil versus the V-PDB (Vienna Pee Dee Belemnite) standard.

Analytical uncertainties were ∼1 % for He and Ne, 1.5 % for ³He/⁴He, and 0.2‰ for δ¹³C (CO_2_).

## Limitations

The Multi-GAS cannot be used to measure the compositional features of gas emissions with very low fluxes (below the free flow value of the Multi-GAS pump, which is 1400 ml/min). Sampling in copper tubes is adequate for noble gas sampling, but it also means that some gas species that react with the copper tube, e.g. H_2_S are lost. Such components are either measured in situ (e.g. H_2_S measurement with Multi-GAS, or using other sampling methods, e.g. Giggenbach bottles).

## Ethics Statement

The authors confirm that they have read and followed the ethical requirements for publication in Data in Brief. This work does not involve human subjects, animal experiments, or any data collected from social media platforms.

## CRediT Author Statement

**B.M.K.**: conceptualization, data curation, funding acquisition, investigation, project administration, methodology, writing – original draft. **R. Sz.**: visualization, investigation. **A.C.**: conceptualization, investigation, writing - review and editing. **P.R.**: visualization, investigation. **T.M.T.**: conceptualization, investigation, writing - review and editing. **L.P.**: methodology, writing - review and editing. **J.O.**: methodology. **A.A.**: methodology, writing - review and editing. **F.G.**: methodology. **Sz.H.**: conceptualization, investigation, writing - review and editing.

## Data Availability

Earth/ChemResearch data in repository (Original data)

## References

[1] Fischer T.P., Aiuppa A. (2020). AGU Centennial Grand Challenge: volcanoes and deep carbon global CO2 emissions from subaerial volcanism-recent progress and future challenges. Geochem. Geophys. Geosystems.

[2] Kis B.M., Caracausi A., Palcsu L., Baciu C., Ionescu A., Futó I., Sciarra A., Harangi S. (2019). Noble gas and carbon isotope systematics at the seemingly inactive Ciomadul Volcano (Eastern-Central Europe, Romania): evidence for volcanic degassing. Geochem. Geophys. Geosystems.

[3] Kis B.M., Szalay R., Aiuppa A., Bitetto M., Palcsu L., Harangi S. (2022). Compositional measurement of gas emissions in the Eastern Carpathians (Romania) using the Multi-GAS instrument: approach for in situ data gathering at non-volcanic areas. J. Geochem. Explor..

[4] Kis B.M., Szalay R., Caracausi A., Randazzo P., Tóth M.T., Palcsu L., Orsovszki J., Aiuppa A., Grassa F., Harangi Sz. (2025). Location, chemical and isotopic composition of free bubbling gases collected in the Eastern Carpathians. Rom. EarthChem Repos. Interdiscip. Earth Data Alliance (IEDA).

[5] Kis B.M., Szalay R., Caracausi A., Randazzo P., Tóth M.T., Palcsu L., Orsovszki J., Aiuppa A., Grassa F., Harangi Sz. (2025). Geochemistry of CO2-rich gas emissions in the Carpathians: multiscale geological sources and implications for orogenic degassing. Earth-Sci. Rev..

[6] Kotarba M.J., Nagao K. (2008). Composition and origin of natural gases accumulated in the Polish and Ukrainian parts of the Carpathian region: gaseous hydrocarbons, noble gases, carbon dioxide and nitrogen. Chem. Geol..

[7] Palcsu L., Vető I., Futó I., Vodila G., Papp L., Major Z. (2014). In-reservoir mixing of mantle-derived CO_2_ and metasedimentary CH_4_–N_2_ fluids – Noble gas and stable isotope study of two multistacked fields (Pannonian Basin System, W-Hungary). Mar. Petr. Geol..

[8] Papp L., Palcsu L., Major Z., Rinyu L., Tóth I. (2012). A mass spectrometric line for tritium analysis of water and noble gas measurements from different water amounts in the range of microlitres and millilitres. Isot. Env. Health Stud..

[9] Tamburello G., Pondrelli S., Chiodini G., Rouwet D. (2018). Global-scale control of extensional tectonics on CO2 earth degassing. Nat. Commun..

[10] Vaselli O., Minissale A., Tassi F., Magro G., Seghedi I., Ioane D., Szakacs A. (2002). A geochemical traverse across the Eastern Carpathians Romania: constraints on the origin and evolution of the mineral water and gas discharges. Chem. Geol..

[11] Werner, C., Fischer, T.P., Aiuppa, A., Edmonds, M., Cardellini, C., Carn, S., Chiodini, G., Cottrell, E., Burton, M., Shinohara, H., Allard, P., 2019. Carbon dioxide emissions from subaerial volcanic regions: two decades in review. Deep Carbon: Past to Present 188–236.

[12] Zhang M., Xu S., Sano Y. (2024). Deep carbon recycling viewed from global plate tectonics. Nat. Sci. Rev..

